# Response surface optimization of ultrasonic‐assisted extraction of carotenoids from oil palm (*Elaeis guineensis* Jacq.) fronds

**DOI:** 10.1002/fsn3.22

**Published:** 2013-01-04

**Authors:** C. Ofori‐Boateng, K. T. Lee

**Affiliations:** ^1^ Lignocellulosic Research Group School of Chemical Engineering Universiti Sains Malaysia 14300 Nibong Tebal Pulau Pinang Malaysia

**Keywords:** Carotenoids, HPLC–FLD, oil palm fronds, response surface methodology, ultrasonic‐assisted

## Abstract

Oil palm (*Elaeis guineensis* Jacq.) fronds (OPF) are the most abundant oil palm solid wastes that are generated during oil palm agriculture and harvest. Palm oil and some other palm wastes have been reported to contain high concentrations of carotenoids with vital bioactive properties. However, the extraction and quantification of carotenoids from OPF have not been reported. In this study, ultrasonic‐assisted extraction, HPLC–FLD for quantification, and response surface methodology (RSM) for optimization of β‐carotene, lutein, and zeaxanthin from OPF extracts were investigated. The effects of extraction temperature (*X*_1_: 30–70°C), extraction time (*X*_2_: 10–50 min), and solvent–sample ratio (*X*_3_: 10–50 mL/g) on the recovery of β‐carotene (*Y*_1_), lutein (*Y*_2_), and zeaxanthin (*Y*_3_) were investigated using three‐level Box–Behnken design (BBD) experiment. At a desirability of 1, the optimum extraction conditions for β‐carotene (30.14°C, 37.11 min, and 23.18 mL/g), lutein (30.00°C, 39.09 min, and 19.24 mL/g), and zeaxanthin (30.09°C, 36.76 min, and 22.38 mL/g) yielded carotenoid concentrations of 17.95 μg/g dry weight (DW), 261.99 μg/g DW, and 29.99 μg/g DW, respectively.

## Introduction

Currently, palm oil is found to be the most consumed vegetable oil (of the 17 major edible oils) in the world. The oil palm industry therefore keeps expanding in capacity resulting in the generation of over 21.63 kg/ha of palm wastes every year (Yusoff 2006), which has a potential phytochemical production capacity of about 220 kg. Oil palm fronds (OPF) are the most abundant palm wastes generated in the oil palm plantation after pruning and harvesting. In Malaysia for instance, in 2010 and 2011, about 54.17 million tonnes and 54.24 million tonnes of OPF were generated from the oil palm industry, respectively, as against about 36 million tonnes in 2004 (Wan Zahari et al. 2004). However, these wastes are improperly disposed of, hence creating environmental problems. Utilization of these wastes for value‐added bioproducts such as bioethanol and phytochemicals would not only save the environment but also add economic value to the oil palm, hence the move toward sustainable palm oil production. Palm oil is found to contain high quantities of carotenoids and it is believed that other parts of the palm tree (and palm wastes) may contain significant amount of these plant chemicals, which could be tapped for health purposes.

Carotenoids are isoprenoids that consist of 40 carbon atoms and are the most important photosynthetic pigments, which possess the ability to protect chlorophyll and thylakoid membrane from photo oxidative damage (Bauerfeind et al. 1971). Of all the provitamin A carotenoids that exist, β‐carotene is the most important when it comes to the protection against chronic diseases such as cancer, diabetes, cardiovascular diseases, etc. (Ford et al. 1999; Perera and Yen 2007). Lutein and zeaxanthin are the major pigments present in the macular region of the retina and are responsible for protection against age‐related macular degeneration. Thus, diets containing lutein and zeaxanthin may help reduce these health‐related problems of the eye (Krinsky et al. 2003). This study therefore aims at optimizing the extraction conditions and quantification of carotenes and xanthophylls from OPF. This research is the first to report the presence of carotenoids from OPF; thus, a useful basis for adding economic value to the oil palm as major parts of their wastes could be utilized for medicinal foods.

In order to effectively quantify bioactive substances from plants, especially lignocellulosic materials, the appropriate extraction method must be employed to conserve resources.

Soxhlet extraction and maceration used in solvent extraction are resource‐use (energy and materials) intensive and mostly result in degradation of plant vitamins and lipids (Schmeck and Wenclawiak 2005; Pingret et al. 2012), especially carotenoids as long extraction time is required. In order to shift phytochemical extraction processes to near sustainability as well as offsetting the drawbacks of the conventional extraction methods, new methods like ultrasonic‐assisted extraction (UAE), microwave‐assisted extraction, supercritical fluid extraction, pressurized fluid extraction, etc., are developed and optimized to increase the extraction yields (Wang and Weller 2006). UAE is outstanding in that it is a simple and fast technique, which consumes less energy, time and materials, thus producing more pure products at higher yields (Vinatoru 2001). During sonication, acoustic cavitation produces cavitation bubbles which causes the rupture of the plants' cell walls and eventually allows easy percolation of solvent into the extractable sample (Vinatoru 2001). This method has been applied to extract carotenoids from corn (Ye et al. 2011), tomato (Lianfu and Zelong 2008), *Dunaliella salina* (Macias‐Sanchez et al. 2008), etc., with comparatively high yields. Optimization of UAE of carotenoids from plant materials would therefore help improve the overall process for recovery of bioactive substances. Also, other parameters like ultrasonic frequency and power can be optimized in order to improve the mass transfer rate for efficient extraction of carotenoids. This study is the first of its kind where UAE is applied to extract carotenoids from OPF.

With the focus on UAE, extraction parameters such as ultrasonic temperature, extraction time, sample‐to‐solvent ratio, ultrasonic frequency and power of ultrasonic waves can be varied to obtain higher extraction yields. This can be effectively achieved using response surface methodology (RSM). In this study, ultrasonic bath was used; hence, the ultrasonic frequency and power were fixed and were not considered for optimization. RSM is an effective statistical technique, which explores the relationships between several explanatory variables and one or more response variables with the main aim of optimizing the process (Box and Wilson 1951; Atkinson and Donev 1992; Pompeu et al. 2009). It uses the fitting of polynomial equation to the experimental data to describe the behavior of data sets including interactive effects among the examined variables (Zhong and Wang 2010). RSM optimization is more advantageous than the traditional single parameter optimization in that it saves time and resources (raw materials and solvents) because many experimental runs are carried out during the conventional optimization method (Silva et al. 2007; Ebru and Ozgul 2010; Guo et al. 2010). In this study, the influence of ultrasonic parameters on the concentrations of β‐carotene, lutein, and zeaxanthin in ethanolic extracts of OPF are assessed and optimized using RSM, employing a three‐variable, three‐level Box–Behnken (BBD) design.

## Experimental Procedures

### Chemicals

All chemicals were of highest purity (≥99.0%). Analytical‐grade acetone, ethanol, and high‐performance liquid chromatography (HPLC)‐grade acetonitrile were purchased from Fisher Scientific, U.K. Analytical grade *n*‐hexane (HEX) was obtained from QREC (Asia) Sdn Bhd, Malaysia. 3,5‐Di‐*tert*‐butyl‐4‐hydroxytoluene (BHT), hydrochloric acid (HCl), triethylamine (TEA), l‐ascorbic acid, and ACS‐grade sodium sulfate were also purchased from Sigma‐Aldrich (St. Louis, MO). The internal standards (type II synthetic beta carotene and xanthophylls) were purchased from Sigma‐Aldrich.

### Plant material preparation

Fresh oil palm (*Elaeis guineensis* Jacq.) fronds were obtained from the oil palm plantation at the Engineering campus of Universiti Sains Malaysia. The leaflets were removed leaving the petioles, which were immediately cut into smaller pieces (10–20 mm in length) and washed with tap water. The petioles were further rinsed with deionised water and dried in an oven (Memmert Beschicking‐Loading Modell 100–800) at 40°C overnight. The moisture content was determined on the same day. The dried materials were homogenized using analytical mill (IKA^®^ A11, Retsch, Germany) and then passed through a 500‐μm AS 200 sieve shaker (Retsch, Germany). The samples were packed in polyethylene‐zipped bags and kept below 4°C until solvent extraction.

### Instrumentation

Ultrasonication was carried out in an S 180/(H) Elmasonic (Elma Hans Schmidbauer GmbH & Co., KG, Germany) tank with 327 × 300 × 200‐mm internal dimensions and maximum capacity of 18 L. The sonicator is equipped with a high‐performance 37‐kHz sandwich transducer system with 200‐W ultrasonic power, 800‐W heating power, sweep function for optimized sound field distribution in the cleaning tank, degas function for efficient degassing of the cleaning liquid, a digital timer, and a temperature controller. Evaporation of solvents in extracts were done with a rotary evaporator (LABOROTA 4011‐digital, Heidolph). Chromatographic analyses were performed using Agilent Ultra fast HPLC (Palo Alto, CA) equipped with Agilent 1260 Infinity fluorescent lamp detector (FLD, G1316A), 1260 quaternary pump (G1311B), and ZORBAX Rx‐SIL eclipse plus C18 column (4.6 × 100 mm, 3.5 μm). Data output were analyzed using Agilent LC & LC/MS ChemStation software. Agitation was carried out using a mechanical shaker (Memmert WNB 22) with water bath. Absorbance of standards and samples were measured using a Shimadzu UV‐Visible Spectrophotometer 1601.

### Preparation of calibration standards and curves

Fresh stock standard solutions of β‐carotene, lutein, and zeaxanthin were prepared in chloroform (100 μg/mL). Working standards were made by diluting aliquots from the stock solution (7, 6, 5, 4, 3, 2 mL for β‐carotene; 10, 8, 6, 4, 2 mL for lutein, and 4, 3.8, 3.6, 3.4, 3.2 mL for zeaxanthin) with 20 mL chloroform. Absorbances were measured at 465 nm for β‐carotene and 445 nm for xanthophylls, and the concentrations were calculated using equation (1) (Britton 1995):

(1) $$C_{1}(\mu g/\mathrm{mL})=\frac{\mathrm{ABS}\times 10^{4}}{A_{1\mathrm{cm}}^{1\%}}$$

where *C*_1_, ABS, and $A_{1\mathrm{cm}}^{1\%}$ are concentration of standards, absorbance of standards, and absorption coefficient of β‐carotene and xanthophylls in chloroform (2396 and 2500, respectively) (Britton 1995), respectively. The purity of the standards (eq. (2)) (Britton 1995) was determined using HPLC–FLD by injecting 2 μL of the standard after dilution of 4 mL with 1 mL methanol:

(2) $$\%\mathrm{Purity}=\frac{\mathrm{PA}\times 100}{\mathrm{TA}}$$

where PA and TA are peak area and total area of standards, respectively.

The concentrations calculated with equation (1) were corrected using equation (3) (Britton 1995):

(3) $$C(\mu g/\mathrm{mL})=\frac{C_{1}(\mu g/\mathrm{mL})\times \%\mathrm{Purity}}{100}$$

where *C* is the final concentration used for calibration curve preparation.

Working standard solutions were wrapped in aluminum foil and kept below 4°C. These solutions were standardized twice in a week before injection into HPLC instrument. The working solutions were vacuum filtered through a 0.2‐μm cellulose membrane and immediately injected into the HPLC equipment. The concentrations of the working solutions were used to construct calibration curves by linear regression (*R* ^2^: 0.9930, 0.9957, and 0.9964 for β‐carotene, lutein, and zeaxanthin, respectively) of the peak area of the individual standards against the standard concentrations. The remaining working solutions were stored in the dark at ≤4°C until further analysis.

### Carotenoids extraction

In order to obtain the solvent suitable to extract high concentrations of carotenoids from palm fronds, three food‐grade solvents (ethanol, *n*‐hexane, and acetone) were used for preliminary extraction. Ethanol, *n*‐hexane, and acetone are cheap and reusable food‐compatible biosolvents, which have the ability to stabilize against oxidation, thus widely used in the recovery of carotenoids from plant tissues.

Briefly, the dried palm fronds (10 g) were mixed with 0.1 g l‐ascorbic acid already dissolved in the solvent (100 mL), placed in the shaker bath, and shaken continuously for 3 h at 40°C. The supernatants were collected after filtering and the residues were subjected to additional three rounds of extraction when the filtrate was colorless. The three solvent extracts were analyzed by HPLC–FLD immediately after extraction.

#### Alkaline hydrolysis

A modified method of Howe and Tanumihardjo (2006) and Bendahou et al. (2007) was used for alkaline hydrolysis and extraction of palm fronds' carotenoids. Briefly, the homogenized dried sample (10 g) was mixed with l‐ascorbic acid in ethanol (0.2 g in 5 mL ethanol) and 15 mL of aqueous KOH (60% w/v), and then placed in a water bath shaker at 50°C for 10 min with continuous shaking. The reaction was halted by placing in cold water for some time and then filtered through Whatman filter paper no. 1. The supernatant was therefore subjected to triplicate extraction with ethanol at 50°C for 30 min. The organic layer after collection was concentrated in vacuo with rotary evaporator at 30 ± 2°C and reconstituted in ethanol before HPLC analysis. The extracts were immediately wrapped with foils and put in the freezer until further analysis. Experiments were carried out under dim light to avoid light‐induced carotenoid degradation and photoisomerization.

#### Ultrasonic‐assisted extraction

Ultrasonication was performed in triplicate following a modified method of Jing et al. (2008). Homogenized dried sample (10 g) was mixed with 0.2 g l‐ascorbic acid dissolved in 100 mL ethanol in a volumetric flask (varying sample‐to‐solvent ratio from 1:10 to 1:50 g/mL). The mixture was placed in the ultrasound cleaning bath for a specified time (varying from 10 to 50 min) at constant temperature (varying from 30 to 70°C). The mixture was filtered and the residue used for triplicate extraction. The supernatants were pooled together and used for HPLC analysis immediately after filtering through a 0.45‐μm cellulose membrane filter.

### HPLC–FLD analysis

Sample injections were done using 1260 manual injector at 20 μL per injection. β‐carotene, lutein, and zeaxanthin contents in OPF were analyzed using Agilent Ultra fast HPLC (Palo Alto, CA) with the following conditions:

Detector: fluorescent lamp (FLD)

Mobile phase: methanol:acetonitrile:TEA (85:14:1 v/v/v)

Emission wavelength: 400 nm

Excitation wavelength: 550 nm

Flow rate: 0.4 mL/min

Run time: 15 min.

**Figure 1:**
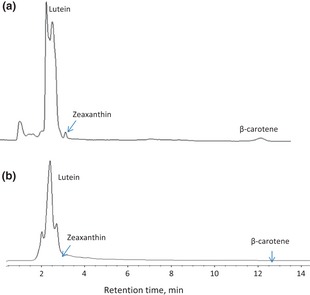
HPLC–FLD chromatograms of ethanolic extract (a) and *n*‐hexane extract (b) of OPF. HPLC, high‐performance liquid chromatography; FLD, fluorescent lamp detector; OPF, oil palm fronds.

With these conditions, lutein, zeaxanthin, and β‐carotene standards eluted at approximately 2.5, 3.4, and 12.8 min, respectively (Fig. 1). The individual carotenoid concentrations were quantified based on the congruence of retention times and peak areas relative to those of the standards using the standard calibration curves. Results were evaluated based on analyses done in triplicate and were expressed as mean values.

### Experimental design and statistical analysis

A three‐variable, three‐level BBD (Box and Wilson 1951; Box and Behnken 1960; Ferreira et al. 2007; Wang et al. 2008) was employed in optimizing the extraction conditions for high recovery of carotenoid concentrations from OPF. Three independent variables namely extraction temperature (*X*_1_, °C), extraction time (*X*_2_, min), and solvent‐to‐sample ratio (*X*_3_, mL/g) were coded at three levels (−1, 0, +1) and used to obtain the coefficients of the quadratic polynomial model using the aid of the software Design‐Expert 8.0.7.1 (State‐Ease, Inc., Minneapolis, MN). A total of 17 experiments (Table 2) were designed and run in triplicate with the average concentrations of β‐carotene, lutein, and zeaxanthin taken as the responses *Y*_1_, *Y*_2_, and *Y*_3_, respectively. The quality of the fitted model was expressed by the coefficient of determination (*R* ^2^) and its statistical significance was checked by *F*‐test and *P*‐value test. The statistical analysis of the model was performed in the form of analysis of variance (ANOVA). Regression analysis was performed and fitted into the empirical second‐order polynomial model for the experimental data as shown in equation (4):

(4) $$Y=\beta_{0}+\sum_{i=1}^{3}\beta_{i}X_{i}+\sum_{i=1}^{3}\beta_{\mathrm{ii}}X_{i}^{2}+\sum_{i=1}^{2}\sum_{j=i+1}^{3}\beta_{\mathrm{ij}}X_{i}X_{j}$$

where *β*_0_, *β*_*i*_, *β*_*ii*_ *,* and *β*_*ij*_ are the intercept, regression coefficients of the linear, quadratic, and interaction terms of the model, respectively, while *X*_*i*_ and *X*_*j*_ are the independent variables and *Y* is the dependent variable (concentrations of the carotenoids).

## Results and Discussions

### Choice of solvent and extraction method for optimization

**Figure 2:**
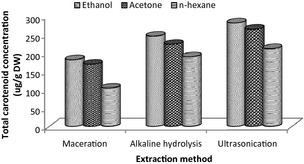
Effect of solvent type and extraction method on total carotenoid concentrations of oil palm fronds (OPF).

In selecting the appropriate solvent for the extraction of carotenoids from OPF, *n*‐hexane (nonpolar solvent), acetone (polar aprotic solvent), and ethanol (polar protic solvent) were used to extract carotenoids from OPF, first using the conventional maceration method at 40°C for 3 h. Ethanolic extract had the highest concentrations of total carotenoids of compositions 6.24 ± 0.04, 126.83 ± 2.28, and 20.99 ± 0.12 μg/g dry weight (DW) for β‐carotene, lutein, and zeaxanthin, respectively. Acetone extracts contained 5.68 ± 0.02 μg/g DW β‐carotene, 118.27 ± 3.51 μg/g DW lutein, and 17.88 ± 0.89 μg/g DW zeaxanthin, which were quite higher than those found in *n*‐hexane extracts (9.30 ± 0.06, 60.83 ± 1.38, and 11.45 ± 0.23 μg/g DW for β‐carotene, lutein, and zeaxanthin, respectively) (Fig. 2). Next, two extraction methods (alkaline hydrolysis and UAEs) were employed to determine the method which is able to extract more carotenoids. UAE with ethanol recorded high concentration values of total carotenoids (13.21 ± 0.23, 220.55 ± 0.56, and 23.07 ± 0.94 μg/g DW for β‐carotene, lutein, and zeaxanthin, respectively), although closer to those for alkaline hydrolysis (11.94 ± 0.18, 199.73 ± 0.39, and 21.05 ± 0.06 μg/g DW for β‐carotene, lutein, and zeaxanthin, respectively); hence, UAE method was optimized for carotenoid extraction from ethanolic extracts of the OPF.

Ethanolic extract contained more lutein (126.83 μg/g DW) and zeaxanthin (20.99 μg/g DW), but low concentration of β‐carotene (6.24 μg/g DW) compared with those for acetone and *n*‐hexane. However, *n*‐hexane extract recorded the highest concentration of β‐carotene (9.30 μg/g DW) compared with the other solvent extracts, probably because carotenes are nonpolar and highly attracted to nonpolar solvents. This clearly shows that, ethanol is able to efficiently extract xanthophylls (polar substances) compared with carotenes (nonpolar). The concentrations of β‐carotene, lutein, and zeaxanthin in acetone extract were found to differ significantly (*P* < 0.05) from those for ethanolic extracts. Generally, the total lutein, β‐carotene, and zeaxanthin concentrations in ethanolic extract were more than those found in *n*‐hexane and acetone extracts. The concentrations of carotenoids from the UAE and alkaline hydrolysis extraction methods were about twice of those present in OPF using maceration. Also, by using ethanol in UAE and alkaline hydrolysis extraction, the former was found to extract more carotenoids (total concentration of lutein, zeaxanthin, and β‐carotene), hence used for optimization in this study.

#### Selection of solvent:sample ratio range for UAE

**Figure 3:**
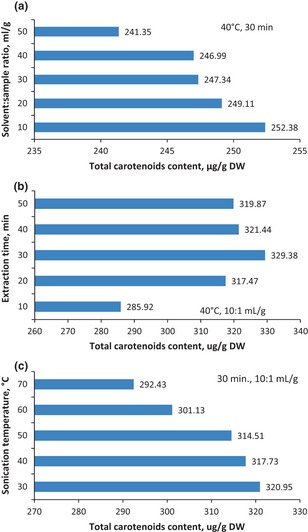
Effect of UAE conditions on total carotenoid content of ethanolic extract of OPF. UAE, ultrasonic‐assisted extraction; OPF, oil palm fronds.

Preliminary experiments of UAE of carotenoids from ethanolic extracts of OPF were performed in order to determine the required ratio of volume of ethanol (mL) to OPF weight (g). Range of ratios (10:1, 20:1, 30:1, 40:1, and 50:1 mL/g) were run in triplicate, while extraction temperature and extraction time at 40°C and 30 min, respectively, were held constant. After analyzing the extracts by spectroscopy, solvent:sample ratio was found to have effect on the concentrations of total carotenoids (Fig. 3a). The maximum concentrations were achieved at 10:1 mL/g for all the carotenoids studies; hence, a ratio of 10:1–20:1 mL/g would be favorable for UAE.

#### Selection of extraction time for UAE of carotenoids from OPF

Carotenoid contents are found to be affected by extraction temperature and at high temperatures, they tend to degrade. UAE of the carotenoids was carried out using extraction time range of 10, 20, 30, 40, and 50 min, while the extraction temperature and solvent:sample ratio were kept at 40°C and 10:1 mL/g. Extraction time of 30 min resulted in the highest carotenoid concentrations (Fig. 3b); thus, times between 20 and 40 min would be suitable for the extraction.

#### Selection of extraction temperature for UAE

In choosing the range of extraction temperature for UAE, the solvent:sample ratio of 20:1 mL/g and extraction time of 30 min were used to run triplicate experiments with extraction temperature range of 30, 40, 50, 60, and 70°C. The carotenoid concentrations were highest at 40°C (Fig. 3c). Thus, the temperature range of 30–50°C was considered optimal for this preliminary experiment. Conventional maceration and soxhlet extraction requires high temperatures (over 70°C) for optimal carotenoid yields as opposed to UAE (Guo et al. 2010).

### Optimization of carotenoid extraction from OPF: analysis for Box–Behnken experiment

#### Response surface models fitting

The effects of three main variables on UAE of carotenoids from palm fronds was simultaneously investigated using a three‐factor design with three levels for each factor (low [−], medium [0], and high [+]). The main aim of the optimization process was to maximize the UAE of β‐carotene (*Y*_1_), lutein (*Y*_2_), and zeaxanthin (*Y*_3_) concentrations in ethanolic extracts of OPF. In optimizing the UAE, the effect of three main independent variables namely ultrasonic temperature of 30–70°C, extraction time of 10–50 min, and solvent:sample ratio of 10:1–50:1 mL/g was simultaneously studied using three‐factor (*X*_1_, *X*_2_, *X*_3_), three levels for each factor (−1, 0, +1) (Table 1) to determine the responses (concentrations of β‐carotene, lutein, and zeaxanthin), which resulted in 17 experiments. The data obtained in the Box–Behnken experiment were converted into second‐order polynomial equation with three independent variables and three responses (*Y* values) as described by equations (5), (6), (7) for β‐carotene, lutein, and zeaxanthin, respectively:

(5) $$\begin{array}{cc} Y_{1} & =+16.87-1.62X_{1}+0.67X_{2}-0.40X_{3}-0.77X_{1}X_{2}\\ & \;+0.27X_{1}X_{3}-0.32X_{2}X_{3}-0.83X_{1}^{2}-2.66X_{2}^{2}-1.27X_{3}^{2} \end{array}$$

(6) $$\begin{array}{cc} Y_{2} & =+258.40-14.31X_{1}+7.47X_{2}-2.31X_{3}-7.87X_{1}X_{2}\\ & \;-1.66X_{1}X_{3}-0.052X_{2}X_{3}-9.05X_{1}^{2}-29.00X_{2}^{2}-10.39X_{3}^{2} \end{array}$$

(7) $$\begin{array}{cc} Y_{3} & =+28.88-1.36X_{1}+0.91X_{2}-0.51X_{3}-1.20X_{1}X_{2}\\ & \;+0.47X_{1}X_{3}-0.005X_{2}X_{3}-0.87X_{1}^{2}-2.48X_{2}^{2}-1.23X_{3}^{2} \end{array}$$

**Table 1:** Observed and predicted values of carotenoid concentrations obtained by Box–Behnken experiment

Factor	Codes	Levels		
		Low (−1)	Medium (0)	High (+1)
Temperature (°C)	*X*_1_	30	50	70
Time (min)	*X*_2_	10	30	50
Solvent:sample ratio	*X*_3_	10	30	50

Carotenoids concentration, μg/g DW									
Standard order	*X*_1_	*X*_2_	*X*_3_	Observed value			Predicted value		
				β‐carotene	Lutein	Zeaxanthin	β‐carotene	Lutein	Zeaxanthin
1	30 (−1)	10 (−1)	30 (0)	12.99	212.97	24.29	13.55	219.33	24.77
2	70 (+1)	10 (−1)	30 (0)	12.17	211.64	24.63	11.87	206.43	24.45
3	30 (−1)	50 (+1)	30 (0)	16.14	244.78	28.82	16.44	249.99	29.00
4	70 (+1)	50 (+1)	30 (0)	12.22	211.99	24.35	11.66	205.63	23.87
5	30 (−1)	30 (0)	10 (−1)	17.95^a^	261.99^b^	29.99^c^	17.06	253.92	29.12
6	70 (+1)	30 (0)	10 (−1)	13.32	225.11	25.67	13.29	228.60	25.45
7	30 (−1)	30 (0)	50 (+1)	15.67	256.11	26.94	15.71	252.62	27.16
8	70 (+1)	30 (0)	50 (0)	12.12^a^	212.61^b^	24.51^c^	13.02	220.68	25.38
9	50 (0)	10 (−1)	10 (−1)	12.01	212.08	24.37	12.35	213.80	24.77
10	50 (0)	50 (+1)	10 (−1)	13.74	225.97	25.89	14.34	228.83	26.59
11	50 (0)	10 (−1)	50 (+1)	12.78	212.15	24.44	12.19	209.29	23.75
12	50 (0)	50 (+1)	50 (+1)	13.21	225.83	25.98	12.88	224.12	25.58
13	50 (0)	30 (0)	30 (0)	16.84	258.28	28.99	16.87	258.40	28.88
14	50 (0)	30 (0)	30 (0)	16.87	257.77	28.76	16.87	258.40	28.88
15	50 (0)	30 (0)	30 (0)	16.85	258.79	28.98	16.87	258.40	28.88
16	50 (0)	30 (0)	30 (0)	16.88	258.97	28.88	16.87	258.40	28.88
17	50 (0)	30 (0)	30 (0)	16.89	258.17	28.79	16.87	258.40	28.88

a Values were considered outliers, thus were not regarded when describing the model for β‐carotene.

b Values were considered outliers, thus were not regarded when describing the model for lutein.

c Values were considered outliers, thus were not regarded when describing the model for zeaxanthin.

The predicted and observed (experimental) values were close to each other (Table 1), making the models precisely adequate. By applying ANOVA for the three regression equations (5), (6), (7), the models were found to be significant (*P* < 0.05), thus very useful in predicting the effects of the three different level factors on carotenoid concentrations for all the three responses (Table 2). However, for β‐carotene and zeaxanthin, solvent‐to‐sample ratio (*X*_3_) as well as all the interaction parameters (*X*_1_ *X*_2_, *X*_1_ *X*_3_, *X*_2_ *X*_3_) were insignificant (*P* > 0.05). All the interaction parameters for lutein were also insignificant (*P* > 0.05) and not the linear‐term coefficients (*X*_1_, *X*_2_, *X*_3_) and quadratic‐term coefficients (*X*_1_^2^, *X*_2_^2^, *X*_3_^2^) (Table 2). The models also showed that the extraction temperatures were the most significant single parameter which influenced the sonication of OPF for all the considered carotenoids, followed by extraction times and solvent‐to‐sample ratios. The predicted and observed coefficients of determination (*R* ^2^) values for the above regressions were close to each other (Table 2), indicating that the model adequately fits the real relationship between the parameters chosen in this study. The “fitness” of the models was studied using the lack‐of‐fit test (*P* > 0.05), which must be insignificant to show the suitability of the models to predict the variations correctly. The *P*‐values of the lack‐of‐fit for the models are 0.0611, 0.0512, and 0.0506 for β‐carotene, lutein, and zeaxanthin, respectively. The results again indicate that the effect of all the independent variables were major contributors to the carotenoid concentrations of OPF by UAE.

**Table 2:** Analysis of variance for response surface methodology quadratic model for carotenoid concentrations from ethanolic extracts of oil palm fronds

Carotenoid	Source	Sum of squares	Degree of freedom	Mean squares	*F*‐value	*P*‐value
β‐carotene	Model	71.70	9	7.97	16.67	0.0006
	Sonication temperature *X*_1_	20.87	1	20.87	43.65	0.0003
	Extraction time, *X*_2_	3.59	1	3.59	7.51	0.0289
	Solvent:sample ratio, *X*_3_	1.31	1	1.31	2.75	0.1415
	*X*_1_^2^	2.89	1	2.89	6.04	0.0436
	*X*_2_^2^	29.75	1	29.75	62.23	0.0001
	*X*_3_^2^	6.82	1	6.82	14.27	0.0069
	*X*_1_ *X*_2_	2.40	1	2.40	5.03	0.0599
	*X*_1_ *X*_3_	0.29	1	0.29	0.61	0.4604
	*X*_2_ *X*_3_	0.42	1	0.42	0.88	0.3784
	Residual	3.35	7	0.48		
	Correlation total	75.04	16			
	*R* ^2^	0.9554				
	Adjusted *R* ^2^	0.8981				
	Adequate precision	10.174^a^				
Lutein	Model	7061.11	9	784.57	17.55	0.0005
	Sonication temperature *X*_1_	1638.78	1	1638.78	36.65	0.0005
	Extraction time, *X*_2_	445.96	1	445.96	9.97	0.0160
	Solvent:sample ratio, *X*_3_	42.55	1	42.55	0.95	0.3618
	*X*_1_^2^	344.99	1	344.99	7.72	0.0274
	*X*_2_^2^	3540.87	1	3540.87	79.19	0.0001
	*X*_3_^2^	454.47	1	454.47	10.16	0.0153
	*X*_1_ *X*_2_	247.43	1	247.43	5.53	0.0500
	*X*_1_ *X*_3_	10.96	1	10.96	0.25	0.6357
	*X*_2_ *X*_3_	0.011	1	0.011	0.00024	09879
	Residual	312.98	7	44.71		
	Correlation total		16			
	*R* ^2^	0.9576				
	Adjusted *R* ^2^	0.9030				
	Adequate precision	10.288^a^				
Zeaxanthin	Model	68.89	9	7.65	15.46	0.0008
	Sonication temperature *X*_1_	14.80	1	14.80	29.88	0.0009
	Extraction time, *X*_2_	6.68	1	6.68	13.49	0.0079
	Solvent:sample ratio, *X*_3_	2.05	1	2.05	4.14	0.0813
	*X*_1_^2^	3.22	1	3.22	6.51	0.0380
	*X*_2_^2^	25.95	1	25.95	52.39	0.0002
	*X*_3_^2^	6.34	1	6.34	12.81	0.0090
	*X*_1_ *X*_2_	5.78	1	5.78	11.68	0.0112
	*X*_1_ *X*_3_	0.89	1	0.89	1.80	0.2212
	*X*_2_ *X*_3_	0.0001	1	0.0001	0.0002	0.9891
	Residual	3.47	7	0.50		
	Correlation total	72.36	16			
	*R* ^2^	0.9521				
	Adjusted *R* ^2^	0.8905				
	Adequate precision	9.951^a^				

a Adequate precision >4 is considered desirable.

Taking the *F*‐values (16.67, 17.55, and 15.46 for β‐carotene, lutein, and zeaxanthin, respectively) into consideration, the models were significant (Table 2). The predicted residual sum of squares for all the models were 53.51, 4994.09, and 54.82 for β‐carotene, lutein, and zeaxanthin, respectively, which implies that the models fit each point in the design. Coefficient of variation (CV) describes the extent to which the experimental data are dispersed and in the models developed in this study, CV values were 4.73, 2.83, and 2.63% for β‐carotene, lutein, and zeaxanthin, respectively. Low values of the CV (between 1.54% and 9.55%) indicate good precision and reliability of the experiments (Khuri and Cornell 1996; Kuehl 2000; Ahmad et al. 2005); hence, the models are reliable and reproducible. Adequate precision is a measure of the range in predicted response relative to its associated error. It measures the signal‐to‐noise ratio; thus, values of 4 and above are considered desirable (Mason et al. 2003). The adequate precision for all the models (10.174, 10.288, and 9.951 for β‐carotene, lutein, and zeaxanthin, respectively) was desirable (Table 2).

#### Effects of extraction parameters on carotenoid concentrations

The experimental values compared with the predicted ones of carotenoids concentrations obtained with the different combinations of independent variables for β‐carotene (12.12–17.95 μg/g DW and 11.66–17.06 μg/g DW, respectively), lutein (211.61–261.99 μg/g DW and 205.63–258.40 μg/g DW, respectively), and zeaxanthin (24.51–29.99 μg/g DW and 23.75–29.12 μg/g DW, respectively) were close to each other.

Extraction temperature is one of the important factors affecting the extraction of carotenoids from plant materials. At higher temperature (above 30.14°C) with UAE, a lower content of carotenoids are obtained as opposed to the conventional maceration, which releases carotenoids at high temperatures yet with minimal yield. However, carotenoid concentrations were found to decrease at elevated temperatures during the preliminary extraction for solvent selection. Pingret et al. (2012) have also reported the degradation of lipids at high ultrasonic temperatures. The concentration of β‐carotene increased with increase in extraction temperature and extraction time until 30.14°C and 37.11 min, respectively, and then decreased significantly (*P* < 0.05) at temperatures between 40 and 70°C and extraction time from 30 to 50 min. However, there was no significant increase of β‐carotene concentration in the solvent:sample ratio (Fig. 3a–c). Carotenoids are found to degrade at elevated temperatures (Gang and Zora 2001; Meléndez‐Martínez  et al. 2007); thus, this study corresponds to the report by Gu et al. (2008) who also reported an optimum temperature of 30°C for carotenoid extraction. The optimum conditions for β‐carotene extraction were determined to be 30.14°C, 37.11 min, and 23.18 mL/g for an optimal concentration of 17.95 μg/g DW.

Lutein and zeaxanthin followed almost the same trend as β‐carotene. Temperatures above 30.00°C and 30.15°C led to decrease in concentration of lutein and zeaxanthin, respectively. Solvent‐to‐sample ratio did not affect the lutein concentration significantly (*P* > 0.05), but was significantly affected with zeaxanthin concentration (*P* < 0.05). The optimal concentrations of lutein (261.99 μg/g DW) and zeaxanthin (29.99 μg/g DW) were obtained at 30.00°C, 39.07 min and 19.22 mL/g for lutein; and 30.15°C, 36.85 min and 22.74 mL/g for zeaxanthin. It is evident from this study that, generally, lutein, zeaxanthin, and β‐carotene are optimally extracted at low temperatures (30.00–30.15°C), short extraction time (36.85–39.07 min), and low solvent‐to‐sample ratio (19.22:1–23.18:1 mL/g). Extraction temperature was the most significant parameter (*P* < 0.001 for lutein and β‐carotene; *P* < 0.005 for zeaxanthin) in the UAE of carotenoid from OPF.

**Figure 4:**
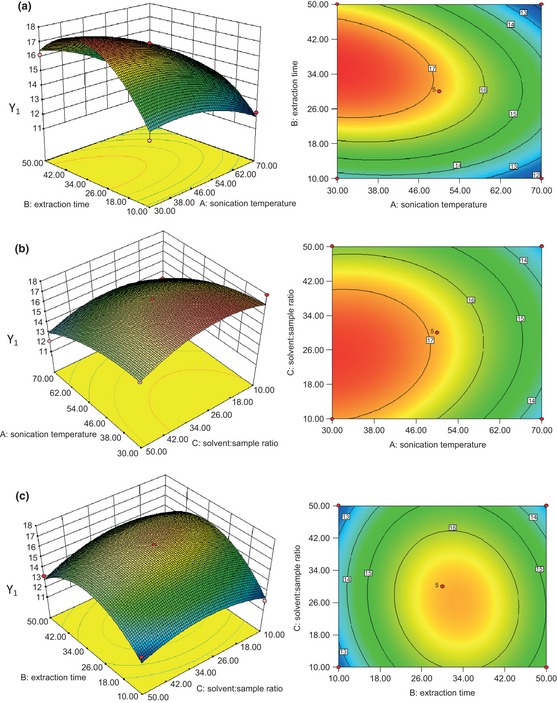
RSM contour and 3D surface plots of the effects of UAE conditions on β‐carotene concentration. (a) Solvent:sample ratio (30:1); (b) time (30 min); (c) temperature (50°C). RSM, response surface methodology; 3D, three‐dimensional; UAE, ultrasonic‐assisted extraction.

**Figure 5:**
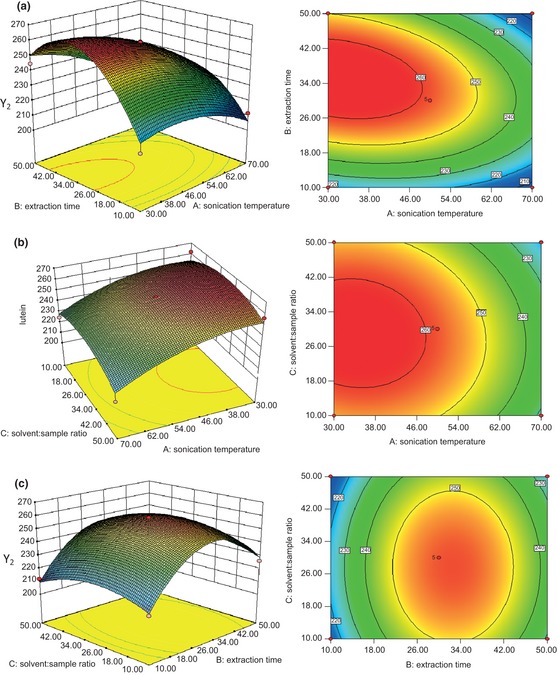
RSM contour and 3D surface plots of the effects of UAE conditions on lutein concentration. (a) Solvent:sample ratio (30:1); (b) time (30 min); (c) temperature (50°C). RSM, response surface methodology; 3D, three‐dimensional; UAE, ultrasonic‐assisted extraction.

**Figure 6:**
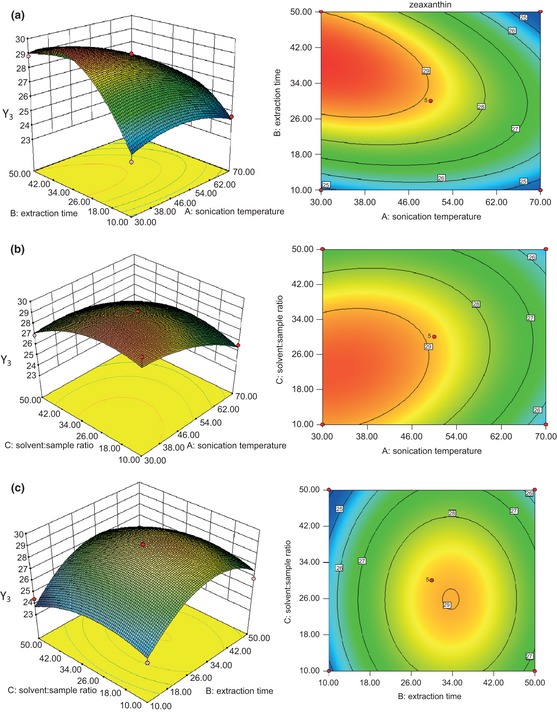
RSM contour and 3D surface plots of the effects of UAE conditions on zeaxanthin concentration. (a) Solvent:sample ratio (30:1); (b) time (30 min); (c) temperature (50°C). RSM, response surface methodology; 3D, three‐dimensional; UAE, ultrasonic‐assisted extraction.

Figures 4, 5, 6 represent the response surface plots (for β‐carotene, lutein, and zeaxanthin, respectively), which explain the effects of the linear, quadratic, and interactive parameters on carotenoid extraction by UAE. The contour and 3D surface plots show the effects of two factors on the response at a time with the other factor kept at zero level.

Figures 4a–c, 5a–c, and 6a–c represent the effects of extraction temperature, extraction time, and their reciprocal interactions on the extraction of β‐carotene, lutein, and zeaxanthin, respectively. An increase in carotenoid concentration was observed with the increase of extraction temperature and extraction time at first, but the carotenoid concentration started to decrease when the extraction temperature and extraction time went past a certain value. Extraction time exhibited an important effect on β‐carotene concentration which was significant (*P* < 0.05), which was opposite to solvent‐to‐sample ratio.

#### Verification of optimized parameters for carotenoid extraction

In order to validate the adequacy of the model equations (eqs. (3), (4), (5), (6), (7)), additional experiments were carried out using the predicted optimized conditions (30°C, 37 min, and 22:1 mL/g for β‐carotene; 30°C, 37 min, and 23:1 mL/g for lutein, and; 30°C, 39 min, and 20:1 mL/g for zeaxanthin) to verify the predicted values for β‐carotene, lutein, and zeaxanthin. These conditions were used to run fresh experiments and the observed mean values (16.94 ± 0.07, 263.22 ± 3.23, and 31.84 ± 0.27 μg/g DW for β‐carotene, lutein, and zeaxanthin, respectively) obtained from these experiments validated the RSM model, which shows that the model was adequate for the extraction process. Predicted carotenoid concentrations obtained from the models were 17.95, 261.99, and 29.99 μg/g DW for β‐carotene, lutein, and zeaxanthin, respectively. The good correlation between these results confirmed that the predicted responses for the models were adequate for reflecting the observed optimization; hence, the models are reproducible. This study shows that RSM is one of the suitable methods to optimize the operating conditions of sonication for extraction of carotenoids from ethanolic extracts of OPF in order to maximize their concentrations.

## Conclusion

The effects of UAE conditions on the concentrations of β‐carotene, lutein, and zeaxanthin from OPF were studied using RSM. The effects of extraction temperature (*X*_1_) and extraction time (*X*_2_) on carotenoid contents were significant (*P* < 0.05), as opposed to solvent‐to‐sample ratio (*X*_3_). Extraction temperature was the most significant parameter (*P* < 0.005) in the UAE of carotenoid from OPF. ANOVA showed a high coefficient of determination, *R* ^2^ = 0.9576, hence establishing a satisfactory adequacy of the models. The optimal concentrations of β‐carotene (17.95 μg/g DW), lutein (261.99 μg/g DW), and zeaxanthin (29.99 μg/g DW) were obtained at 30°C, 36–39 min, and 19–23 mL/g.

## Conflict of Interest

None declared.
